# Potent mouse monoclonal antibodies that block SARS-CoV-2 infection

**DOI:** 10.1016/j.jbc.2021.100346

**Published:** 2021-01-30

**Authors:** Youjia Guo, Atsushi Kawaguchi, Masaru Takeshita, Takeshi Sekiya, Mikako Hirohama, Akio Yamashita, Haruhiko Siomi, Kensaku Murano

**Affiliations:** 1 Department of Molecular Biology, Keio University School of Medicine, Tokyo, Japan; 2 Department of Infection Biology, Faculty of Medicine, University of Tsukuba, Tsukuba, Japan; 3 Transborder Medical Research Center, University of Tsukuba, Tsukuba, Japan; 4 Microbiology Research Center for Sustainability, University of Tsukuba, Tsukuba, Japan; 5 Division of Rheumatology, Department of Internal Medicine, Keio University School of Medicine, Tokyo, Japan; 6 Department of Molecular Biology, Yokohama City University School of Medicine, Yokohama, Japan

**Keywords:** SARS-CoV-2, spike, mouse monoclonal antibody, neutralizing antibody, ACE2, angiotensin-converting enzyme 2, COVID-19, coronavirus disease 2019, ELISA, enzyme-linked immunosorbent assay, ERGIC, endoplasmic reticulum–Golgi intermediate compartment, IF, immunofluorescence, IP, immunoprecipitatio, RBD, receptor-binding domain, RSV, respiratory syncytial virus, SARS-CoV-2, severe acute respiratory syndrome coronavirus 2, SDS, sodium dodecyl sulfate, WB, western blotting

## Abstract

Coronavirus disease 2019 (COVID-19), caused by severe acute respiratory syndrome coronavirus 2 (SARS-CoV-2), has developed into a global pandemic since its first outbreak in the winter of 2019. An extensive investigation of SARS-CoV-2 is critical for disease control. Various recombinant monoclonal antibodies of human origin that neutralize SARS-CoV-2 infection have been isolated from convalescent patients and will be applied as therapies and prophylaxis. However, the need for dedicated monoclonal antibodies suitable for molecular pathology research is not fully addressed. Here, we produced six mouse anti-SARS-CoV-2 spike monoclonal antibodies that not only exhibit robust performance in immunoassays including western blotting, ELISA, immunofluorescence, and immunoprecipitation, but also demonstrate neutralizing activity against SARS-CoV-2 infection to VeroE6/TMPRSS2 cells. Due to their mouse origin, our monoclonal antibodies are compatible with the experimental immunoassay setups commonly used in basic molecular biology research laboratories, providing a useful tool for future research. Furthermore, in the hope of applying the antibodies of clinical setting, we determined the variable regions of the antibodies and used them to produce recombinant human/mouse chimeric antibodies.

The outbreak of coronavirus disease 2019 (COVID-19) caused by severe acute respiratory syndrome coronavirus 2 (SARS-CoV-2) is a threat to global public health and economic development (1, 2). Vaccine and therapeutic discovery efforts are paramount to restrict the spread of the virus. Passive immunization could have a major effect on controlling the virus pandemic by providing immediate protection, complementing the development of prophylactic vaccines (3, 4, 5).

With the development of humanized mouse antibodies and subsequent generation of fully human antibodies by various techniques, monoclonal antibodies have become widely used in therapy and prophylaxis for cancer, autoimmune diseases, and viral pathogens (3). Indeed, a humanized mouse monoclonal antibody neutralizing respiratory syncytial virus (RSV), palivizumab, is widely used in clinical settings prophylactically to protect vulnerable infants (6). In recent years, highly specific and often broadly active neutralizing monoclonal antibodies have been developed against several viruses (3, 7, 8, 9, 10). Passive immunization with a monoclonal antibody is currently under consideration as a treatment for COVID-19 caused by SARS-CoV-2 (4, 11, 12, 13, 14).

Isolation of multiple human neutralizing monoclonal antibodies against SARS-CoV-2 has been reported (15, 16, 17, 18, 19, 20, 21, 22, 23, 24, 25, 26, 27, 28, 29). These antibodies can avoid the potential risks of human–anti-mouse antibody responses and other side effects (30). However, since they are recombinant human antibodies produced in HEK293 cell lines derived from human embryonic kidney, they have a disadvantage compared with conventional hybridoma-produced antibodies in terms of their lot-to-lot quality control and manufacturing costs (31). Instead, monoclonal antibodies produced by hybridomas are secreted into the culture supernatant, thus their production is straightforward and of low cost, and their quality is stable.

In addition to the impact of monoclonal antibodies on therapy and prophylaxis, they significantly impact the characterization of SARS-CoV-2. To overcome the long-term battle with the virus, we need a detailed understanding of the replication mechanisms underlying its life cycle, including viral entry, genome replication, budding from the cellular membrane, and interaction with host immune systems. These essential pieces of information are required for drug discovery, vaccine design, and therapy development. Despite the large number of neutralizing antibodies reported to inhibit infection, there is an overwhelming lack of data on a well-characterized antibody available for basic research techniques such as western blotting (WB), immunofluorescence, and immunoprecipitation to study the viral life cycle.

Here, we established six monoclonal antibodies against the spike glycoprotein of SARS-CoV-2. The trimeric spike glycoproteins of SARS-CoV-2 play a pivotal role in viral entry into human target cells through the same receptor, angiotensin-converting enzyme 2 (ACE2) as SARS-CoV-1 (32). We evaluated these antibodies for application in molecular pathology research. Among them, two antibodies were shown to attenuate the interaction of spike proteins with ACE2 and neutralized infection of VeroE6/TMPRSS2 cells by SARS-CoV-2. Our antibodies will accelerate research on SARS-CoV-2 and lead to new therapies and prophylaxis.

## Results

### Production of six monoclonal antibodies against spike glycoprotein

**Figure 1:**
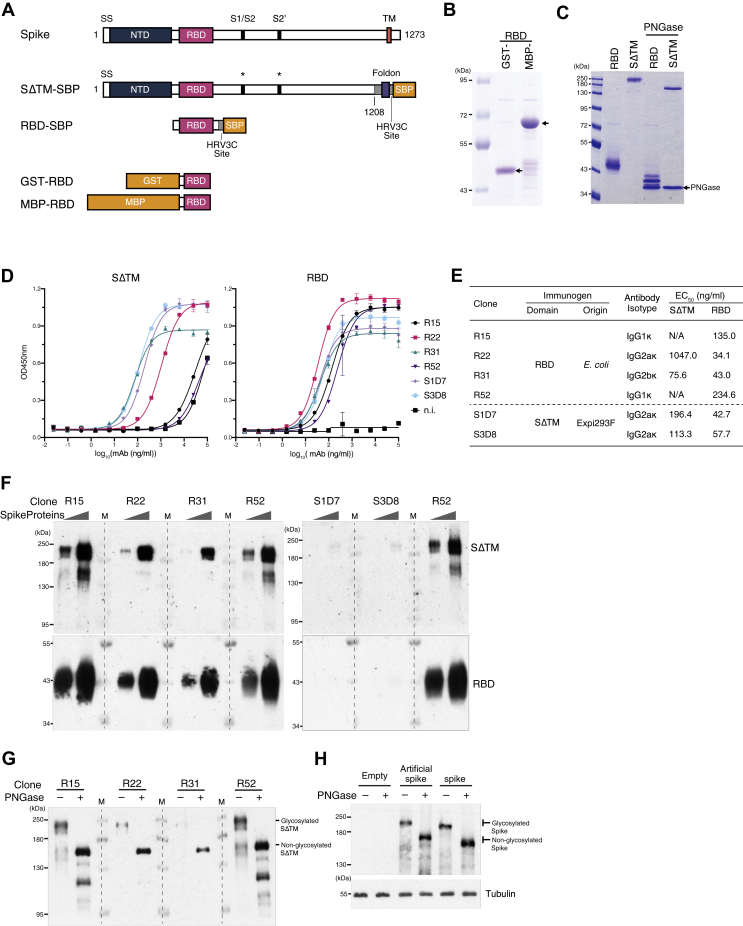
**Production of six monoclonal antibodies against spike protein.***A*, schematic of recombinant proteins used to establish anti-spike antibodies. For mammalian expression constructs (SΔTM-SBP and RBD-SBP), the HRV3C cleavage site was placed upstream of the SBP tag so that the SBP tag could be removed by HRV3C protease treatment after protein purification (Fig. S1*A*). *B*, Coomassie brilliant blue (CBB) staining of recombinant protein purified from *E. coli* expression system. GST-RBD and MBP-RBD appeared as bands of 46 kDa and 62 kDa, respectively. *C*, CBB staining of recombinant proteins purified from the mammalian expression system. The glycosylation of recombinant proteins caused smear bands and a lower migration rate of proteins on SDS-PAGE compared with proteins treated with PNGase. *D*, ELISA-binding affinity of purified monoclonal antibodies to trimeric SΔTM and RBD glycoproteins purified from the mammalian expression system. Error bars indicate standard deviation (n = 3). *E*, summary of isotype and EC_50_ of established monoclonal antibodies. *F*, Western blotting (WB) against SΔTM and RBD glycoproteins (10 or 50 ng per lane) using purified monoclonal antibodies (1 μg/ml in PBS-T). Clone S1D7 and S3D7 could not detect either SΔTM or RBD in WB. *G*, detection of nonglycosylated SΔTM using established monoclonal antibodies. Four clones could detect SΔTM (30 ng per lane), regardless of glycosylation. *H*, detection of spike proteins expressed in 293T cells. Lysates of 293T cells expressing artificial spikes carrying T4 foldon or wild-type spike glycoproteins were separated by SDS-PAGE, followed by WB using antibody R52. Foldon, T4 fabritin trimerization motif; GST, glutathione S-transferase; MBP, maltose-binding protein; n.i., nonimmune mouse IgG; NTD, N-terminal domain; RBD, receptor-binding domain; SΔTM, spike lacking TM domain; SBP, streptavidin-binding peptide; SS, signal peptide; TM, transmembrane domain.

The SARS-CoV-2 spike glycoprotein is a homotrimeric fusion protein composed of two subunits: S1 and S2. During infection, the receptor-binding domain (RBD) on S1 subunit binds to ACE2, resulting in destabilization of the spike protein's metastable conformation. Once destabilized, the spike protein is cleaved into the N-terminal S1 and C-terminal S2 subunits by host proteases such as TMPRSS2 and changes conformation irreversibly from the prefusion to the postfusion state (32, 33, 34), which triggers an infusion process mediated by the S2 region (35, 36). The instability needs to be addressed to obtain high-quality spike proteins for downstream applications. We adopted the design principle reported by Wrapp *et al.* (37), in which the SARS-CoV-2 spike protein was engineered to form a stable homotrimer that was resistant to proteolysis during protein preparation. In our practice, recombinant spike protein RBD and ectodomain were constructed. A T4 fabritin trimerization motif (foldon) was incorporated into the C terminal of the recombinant spike ectodomain to promote homotrimer formation (38) (Fig. 1*A*). Recombinant RBD proteins tagged with GST or MBP were produced using an *E. coli* expression system (Fig. 1*B*). Both recombinant spike protein RBD and ectodomain (SΔTM) were produced using a mammalian expression system that retained proper protein glycosylation equivalent to that observed during virus replication (Figs. 1*C* and S1*A*). Mice were immunized with these recombinant spike proteins to generate antibodies against the SARS-CoV-2 virus, followed by cell fusion to generate a hybridoma-producing antibody. Culture supernatants were prescreened by enzyme-linked immunosorbent assay (ELISA), WB, and immunoprecipitation (IP), and six monoclonal hybridomas were isolated and evaluated.

To characterize these antibodies in detail, they were first purified from the culture supernatant and examined in terms of ELISA and WB performance. Four monoclonal antibodies derived from the antigen produced by *E. coli* (Clones R15, R22, R31, and R52) and two from mammalian cells (S1D7 and S3D8) showed remarkable performance. In the ELISA binding assay, all six clones bound glycosylated RBD with high affinity. When tested against spike glycoprotein (SΔTM), two clones (R15 and R52) could not be distinguished from nonimmune IgG (Fig. 1*D*). We noted that IgG2 subclass members tended to have higher binding affinities. Half maximal effective concentration (EC_50_) required for these antibodies to bind RBD and SΔTM glycoproteins falls at the low hundreds ng/ml (Fig. 1*E*). In WB, where target proteins are reduced and denatured, all clones established by *E. coli* produced-antigens performed well at detecting RBD and SΔTM proteins regardless of glycosylation (Fig. 1*F*, left, and Figs. 1*G* and S1*B*). Among them, clones R15 and R52 showed higher sensitivity in WB. In addition, R52 was capable of detecting not only artificial spike glycoprotein carrying T4 foldon, but also native spike glycoprotein expressed in 293T cells on WB (Fig. 1*H*). However, neither RBD nor SΔTM could be detected by antibody clones established by the mammalian antigen (S1D7 and S3D8) on WB, suggesting a strong preference for intact tertiary structure (Fig. 1*F*, right, see also Fig. S3, *B–D*).

### S1D7 and S3D8 antibodies showed higher performance on IP and IF

**Figure 2:**
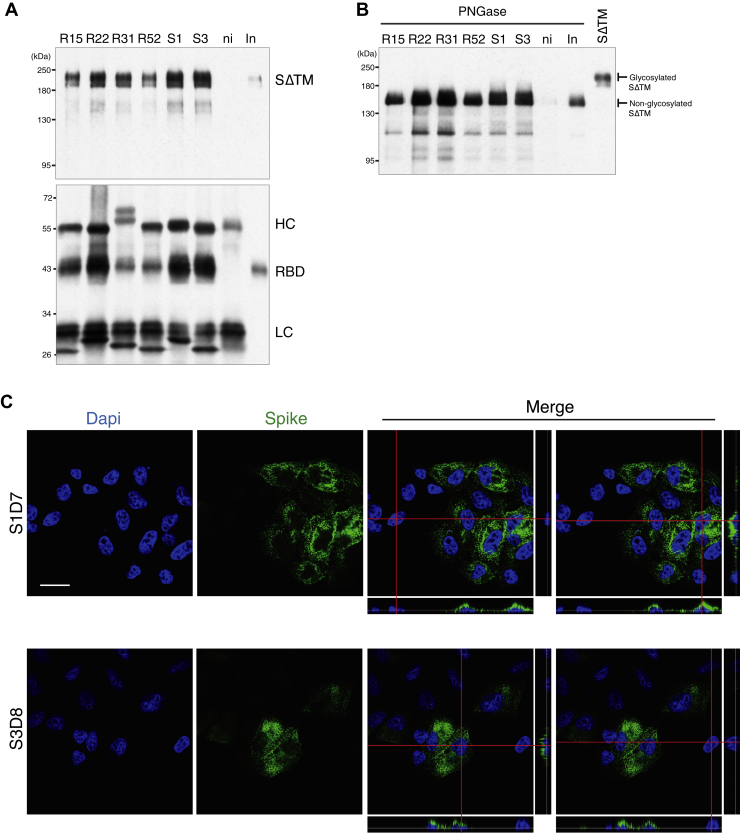
**Application for immunoprecipitation and immunofluorescence.***A*, immunoprecipitation (IP) of trimeric glycosylated spike protein (SΔTM) using established monoclonal antibodies. All clones were capable of pulling down RBD and spike glycoprotein. Higher IP efficiency of spike glycoprotein was observed in clones R22, R31, S1D7, and S3D8. For RBD glycoprotein, clone R22, S1D7, and S3D8 showed higher IP efficiency. *B*, IP of trimeric spike protein deglycosylated by PNGase F using established monoclonal antibodies. "SΔTM" indicates SΔTM glycoprotein untreated with PNGase F. All clones are capable of pulling down deglycosylated spike protein. Higher IP efficiency was observed in clones R22, R31, S1D7, and S3D8. *C*, immunofluorescence (IF) staining of spike glycoprotein expressed in HeLa cells with monoclonal antibodies S1D7 and S3D8. Spike protein localized on the apical surface of transfected HeLa cells. Scale bar, 30 μm. HC, IgG heavy chain; In, input; LC, IgG light chain; ni, nonimmune mouse IgG; SΔTM, trimeric spike protein without transmembrane domain; S1, S1D7; S3, S3D8.

An antibody capable of recognizing the intact tertiary structure of spike proteins would contribute to research dissecting the molecular mechanism of SARS-CoV-2 infection, especially cell entry, where these proteins play a significant role. The IP activity of antibodies can be correlated with the activity of capturing the native structure of the target protein and neutralizing the infection. We examined the IP performance of our monoclonal antibodies. Although all clones were capable of immunoprecipitating RBD and SΔTM glycoproteins, clones R22, R31, S1D7, and S3D8 demonstrated superior IP efficiency for SΔTM, whereas R22, S1D7, and S3D8 showed higher IP efficiency for RBD glycoprotein (Figs. 2*A* and S2*A*). As shown in Figure 2*B*, our antibodies recognize the spike protein in a glycosylation-independent manner, and the IP efficiencies of R22, R31, S1D7, and S3D8, although mild, outperformed others. Noticeably, although clones S1D7 and S3D8 are not capable of performing WB (Fig. 1*F*), a strong preference for tertiary structure grants them remarkable performance in IP, where RBD and SΔTM glycoproteins were pulled down in their native conformation. Of note, we found that S1D7 and S3D8 could maintain intact IP efficiency under highly stringent experimental conditions where sodium dodecyl sulfate (SDS) was present (Fig. S2*B*).

Next, we examined whether our antibodies could be used in the immunofluorescence assay (IF). An antibody applicable for IP would also have activity in IF. Cellular localization of spike proteins is essential for elucidating the mechanism of packaging and maturation of virions during release from the cellular membrane. We tested our antibodies' performance in IF using HeLa cells overexpressing spike protein with the transmembrane domain. Consistent with their performance in the above-mentioned assays (Fig. 2, *A* and *B*), both S1D7 and S3D8 could detect spike proteins expressed homogeneously on the apical side of HeLa cells with a high signal-to-noise ratio (Figs. 2*C* and S2*C*). However, their localization pattern is different from that observed for SARS-CoV-1 spike proteins, which are exclusively localized in the Golgi during infection (39) (see also Fig. 4*B*). Mouse hepatitis coronavirus spike protein localizes in the endoplasmic reticulum–Golgi intermediate compartment (ERGIC) in a membrane (M) protein-dependent manner (40). We then examined the effect of M protein on cellular localization of spike proteins. As shown in Figure S2, *D* and *E*, M protein did not appear to have any impact on localization of spike proteins in HeLa cells, suggesting that mechanisms of viral assembly in SARS-CoV-2 are different from that of SARS-CoV-1 and mouse hepatitis coronavirus.

### ACE2–spike binding inhibition of the monoclonal antibodies

**Figure 3:**
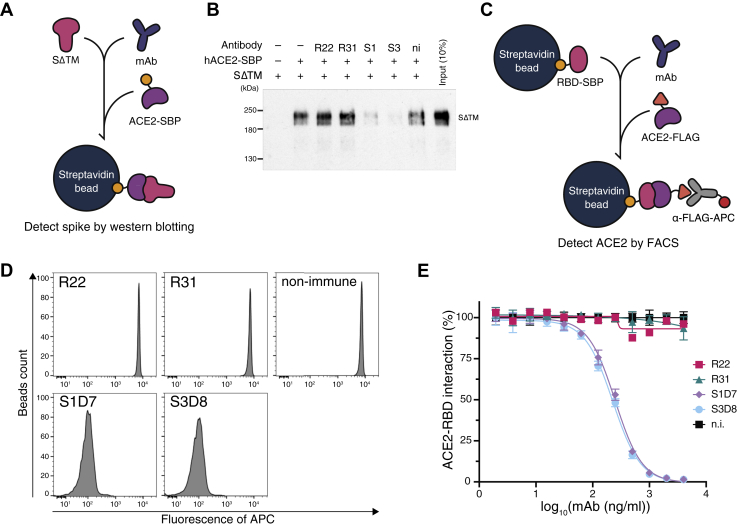
**Inhibition of ACE2–spike interaction by S1D7 and S3D8.***A*, a schematic of the spike pull-down assay designed to evaluate inhibition of ACE2–spike binding by monoclonal antibody. Spike glycoprotein lacking TM domain (SΔTM) was mixed with a monoclonal antibody. ACE2-SBP was applied to capture SΔTM onto streptavidin beads competitively. Captured SΔTM was detected by WB as a measurement of the antibody's inhibitory ability. *B*, WB of spike pull-down assay using antibody R52. In the presence of clones S1D7 and S3D8, ACE2 was not able to pull down SΔTM. *C*, schematic of bead-based neutralization assay designed to quantify inhibition of ACE2–RBD binding by monoclonal antibody. RBD-SBP glycoprotein immobilized on streptavidin beads was mixed with a monoclonal antibody. ACE2-FLAG was applied to bind competitively with RBD. ACE2–RBD binding was quantified by measuring the signal given by an anti-FLAG antibody conjugated with APC fluorophore using FACS. *D*, one set of representative FACS results of a bead-based neutralization assay in the presence of 4 μg/ml monoclonal antibodies. Clones S1D7 and S3D8 significantly inhibited ACE2-RBD interaction, shown as lowered fluorescence intensity of APC. *E*, binding profiles of potent neutralizing antibodies. Error bars indicate standard deviation (n = 3). Clones R22 and R31 showed no inhibition of ACE2-RBD binding, while S1D7 and S3D8 inhibited ACE2-RBD interaction at lower ng/ml levels. ni, nonimmune mouse IgG; S1, S1D7; S3, S3D8.

The manner in which antibodies bind and pull down spike glycoproteins in an IP experiment resembles the process of antibody-mediated neutralization, where spike–ACE2 interaction is intercepted by competitive binding between neutralizing antibodies and spike glycoprotein. We then examined whether they were capable of inhibiting spike–ACE2 binding or even neutralizing SARS-CoV-2 infection. First, we performed a spike pull-down assay in which the spike glycoprotein was pulled down by ACE2 in the presence of monoclonal antibodies (Fig. 3*A* and S3*A*). Clones S1D7 and S3D8 clearly inhibited spike–ACE2 binding, as shown by the dimmed spike signal in WB (Fig. 3*B*). To quantify the inhibition ability, we performed a bead-based neutralization assay by measuring the amount of ACE2 bound to RBD beads after blocking with monoclonal antibodies (Fig. 3*C*). Antibodies R22 and R31 showed no disruption of ACE2–RBD interaction, whereas S1D7 and S3D8 showed robust hindrance of ACE2–RBD binding with IC_50_ values of 248.2 ng/ml and 225.6 ng/ml, respectively (Fig. 3, *D* and *E*). S1D7 and S3D8's abilities to inhibit spike–ACE2 binding was consistent with their superior performance in IP experiments. Four monoclonal antibodies derived from the antigen produced by *E. coli* (Clones R15, R22, R31, and R52) were found to recognize continuous epitope 549-TGVLTESNKKFLPFQQFGRD-568 of spike protein RBD (Fig. S3, *B–D*). In contrast, an epitope of two antibodies from mammalian cells (S1D7 and S3D8) could not be determined (Fig. S3, *B–D*). The fact that they fail to recognize segmented RBD suggests that they recognize an intact tertiary structure of the spike protein.

### S1D7 and S3D8 showed neutralizing activity against SARS-CoV-2

**Figure 4:**
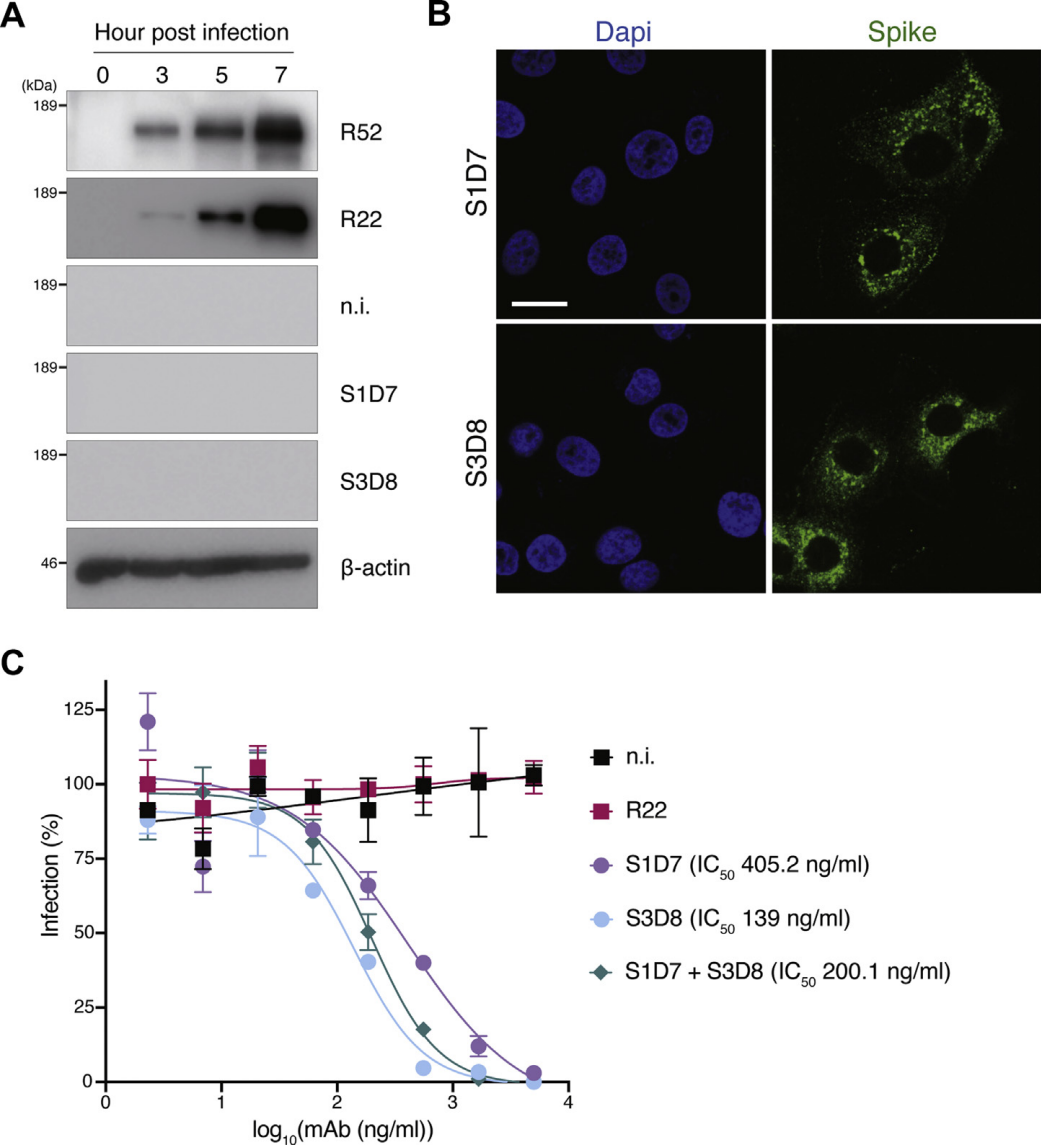
**S1D7 and S3D8 neutralized SARS-CoV-2 infection.***A*, Spike glycoprotein was expressed in VeroE6/TM2 cells during SARS-CoV-2 infection. Spike glycoproteins were detected by western blots using anti-spike antibodies R22 and R52. *B*, immunofluorescence staining of spike glycoprotein expressed in VeroE6/TM2 cells infected with SARS-CoV-2 at 7 h postinfection. Scale bar, 20 μm. *C*, S1D7 and S3D8 are capable of neutralizing live virus infections. Although clone R22 failed to protect VeroE6/TM2 cells from SARS-CoV-2 infection, S1D7 and S3D8 blocked SARS-CoV-2 infection significantly with IC_50_ values of 405.2 ng/ml and 139 ng/ml, respectively. S1D7 and S3D8 cocktail showed intermediate neutralizing activity (200.1 ng/ml). Error bars indicate standard deviation (n = 3).

Next, we asked whether our antibodies inhibit SARS-CoV-2 infection in VeroE6/TMPRSS2 (TM2) cells, which is susceptible to SARS-CoV-2 infection compared with the parental VeroE6 cell line by expressing TMPRSS2 (41). In WB, antibodies R52 and R22, but not S1D7 and S3D8, could detect spike glycoprotein along with the progression of SARS-CoV-2 infection in VeroE6/TM2 cells (Fig. 4*A*). On the other hand, S1D7 and S3D8 were applicable to IF in infected VeroE6/TM2 cells. Spike showed a punctate distribution pattern in the perinuclear region resembling ER and ERGIC (42) (Fig. 4*B*). The subcellular localization of spike resembled that of the N protein in Vero cells infected with SARS-CoV-1 (39), suggesting assembly of SARS-CoV-2 virion in the cytoplasm. We then conducted a live virus neutralization assay to examine whether clones S1D7 and S3D8 inhibit the live virus infection. As expected, although clone R22 failed to protect VeroE6/TM2 from SARS-CoV-2 infection, S1D7 and S3D8 blocked SARS-CoV-2 infection significantly with IC_50_ values of 405.2 ng/ml and 139 ng/ml, respectively, even at relatively high titers of 1500 TCID_50_ (Fig. 4*C* and Table S1). A cocktail of S1D7 and S3D8 showed intermediate neutralizing activity (200.1 ng/ml), suggesting that S1D7 and S3D8 share an inhibitory mechanism.

### Recombinant human/mouse chimeric antibodies R52h and S1D7h are applicable for WB and IF, respectively

**Figure 5:**
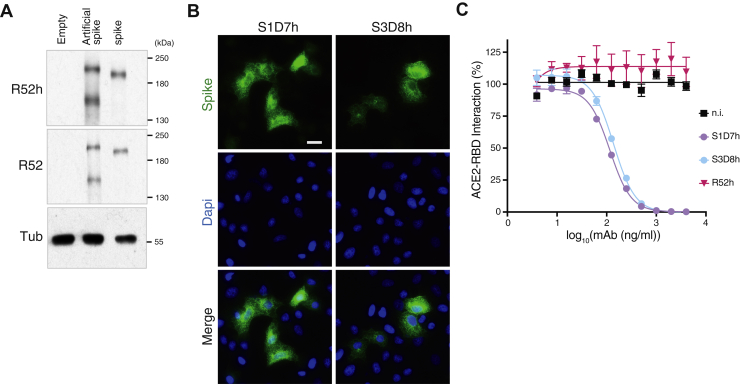
**Recombinant human/mouse chimeric antibodies R52h, S1D7h, and S3D8h.***A*, R52h is applicable for WB. Lysates of 293T cells expressing artificial spikes carrying T4 foldon or wild-type spike glycoproteins were separated by SDS-PAGE, followed by WB using human/mouse chimeric antibody R52h, which was secreted by Expi293F cells. *B*, S1D7h and S3D8h are applicable for IF. Spike glycoprotein expressed in HeLa cells was stained with human/mouse chimeric antibodies S1D7h or S3D8h, which were secreted by Expi293F cells. Scale bar, 20 μm. *C*, Binding profiles of potent neutralizing antibodies. ni, nonimmune mouse IgG. Error bars indicate standard deviation (n = 3).

Our mouse antibodies would not be applicable for use in clinical treatment, if not chimeric and humanized, due to their immunogenicity (30, 43). In the hope of applying the antibodies of clinical settings, the variable regions of the antibodies were determined (Table S2), followed by the production of recombinant antibodies based on plasmid transfection to Expi293 or 293T cell lines. We selected three antibodies from among these and generated humanized chimeric antibodies designated as R52h, S1D7h, and S3D8h by fusing them with the constant region of human IgG1κ for R52, S1D7, and S3D8. R52h was capable of detecting artificial spike glycoprotein carrying T4 foldon, and native spike glycoprotein expressed in 293T cells on WB as well as R52 (Figs. 5*A* and S4*A*). In IF, S1D7h could detect spike proteins expressed in HeLa cells (Figs. 5*B* and S4*B*). Notably, S1D7h and S3D8h showed robust hindrance of ACE2–RBD binding with IC_50_ values of 116.3 ng/ml and 137.2 ng/ml, respectively (Fig. 5*C*).

## Discussion

Emerging SARS-CoV-2 is a global public health threat to society, which is predicted to be long-term for several years (44). Although there are multiple ongoing endeavors to develop neutralizing antibodies, vaccines, and drugs against the virus (45, 46), the lack of adequate, licensed countermeasures underscores the need for a more detailed and comprehensive understanding of the molecular mechanisms underlying the pathogenesis of the virus (47). Fundamental knowledge has significant implications for developing countermeasures against the virus, including diagnosis, vaccine design, and drug discovery. Due to the above reasons, and our experiences with routine antibody productions (48, 49), we have established and characterized mouse monoclonal antibodies that can be used to dissect the molecular mechanism of the virus life cycle. These antibodies would serve as a reliable toolset for basic research investigating the expression profile and subcellular localization of spike glycoprotein during viral entry, replication, packaging, and budding. These antibodies could help to identify novel host factors interacting with spike glycoprotein when used in IP in combination with mass spectrometry. Therefore, advancement in basic research would accelerate the discovery of drugs targeting virus transmission.

Our antibodies, S1D7 and S3D8, have been shown to attenuate the interaction of spike proteins with ACE2 and neutralize infection of VeroE6/TM2 cells by SARS-CoV-2. It is worth noting that although their neutralizing activities (IC_50_ of 405.2 ng/ml and 139 ng/ml) appeared to be lower than those of human antibodies reported previously (Fig. 4*C* and Table S1), the stringency of experimental conditions (relatively high virus titer of 1500 TCID_50_) tend to underestimate neutralizing activities of our antibodies compared with other research groups. Specifically, we used a high multiplicity of live SARS-CoV-2 virus to infect VeroE6/TM2 cells, which are more prone to virus infection than the commonly adopted VeroE6 cell line. Therefore, it is difficult to compare antibody efficacy among them (50). In addition to *in vitro* infection, their neutralizing activity *in vivo* should be examined in animal models that recapitulate SARS-CoV-2 disease. They may be valuable for investigating the mechanism of immune responses to the virus during passive immunization using mouse models for SARS-CoV-2 infection (28, 51, 52, 53, 54, 55). They could show stable performance due to lot-to-lot consistency and act as benchmarks for other antibodies and drug developments.

## Experimental procedures

Experimental procedures are provided as supporting information.

## Data availability

All data are contained within the article.

## Supporting information

This article contains supporting information (56, 57).

## Conflict of interest

The authors declare that they have no conflicts of interest with the contents of this article.
