# Supplementary material for: Potent mouse monoclonal antibodies that block SARS-CoV-2 infection
Source: J Biol Chem. 2021 Jan 30;296:100346. doi: 10.1016/j.jbc.2021.100346 (PMC7846482; doi:10.1016/j.jbc.2021.100346)
Supplement: Supporting information [file mmc1.pdf]

SUPPORTING INFORMATION

for

Potent mouse monoclonal antibodies that block SARS-CoV-2 infection

Youjia Guo[1], Atsushi Kawaguchi[2,3,4], Masaru Takeshita[5], Takeshi Sekiya[2], Mikako Hirohama[2], Akio Yamashita[6], Haruhiko Siomi[1,*], Kensaku Murano[1,*]

[1]Department of Molecular Biology, Keio University School of Medicine, Tokyo, Japan
[2]Department of Infection Biology, Faculty of Medicine, University of Tsukuba, Tsukuba, Japan
[3]Transborder Medical Research Center, University of Tsukuba, Tsukuba, Japan
[4]Microbiology Research Center for Sustainability, University of Tsukuba, Tsukuba, Japan
[5]Division of Rheumatology, Department of Internal Medicine, Keio University School of Medicine, Tokyo, Japan
[6]Department of Molecular Biology, Yokohama City University School of Medicine, Yokohama, Japan

*Corresponding authors: kmurano@keio.jp (K.M.), awa403@keio.jp (H.S.)

Running Title: Mouse anti-SARS-CoV-2 spike monoclonal antibody
Keywords: SARS-CoV-2, spike, mouse monoclonal antibody, neutralizing antibody

Fig. S1

Guo *et al.*

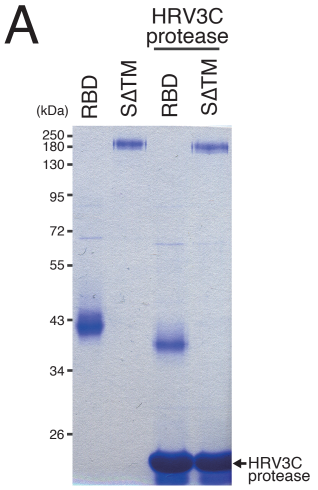

Fig. S2

Guo *et al.*

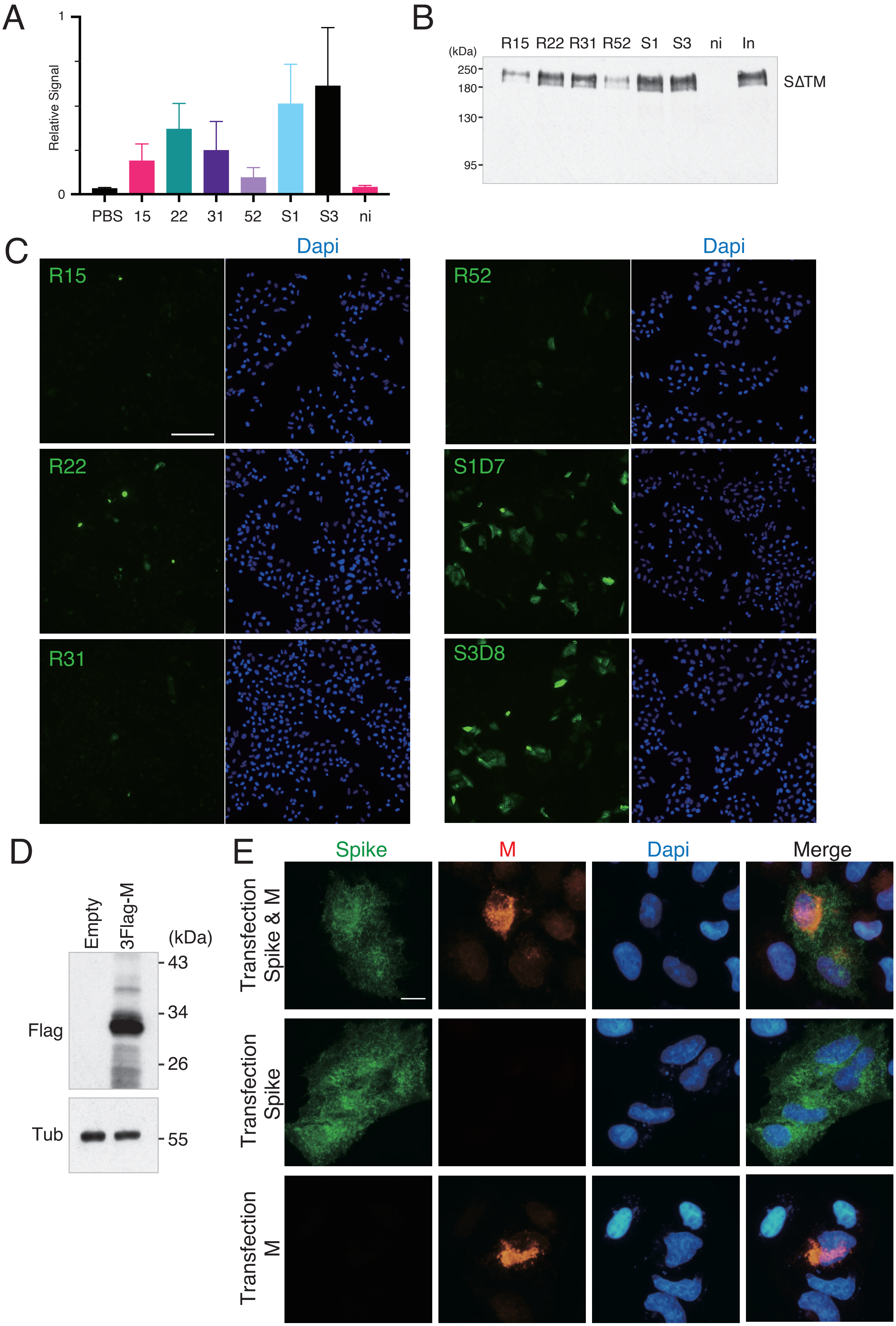

Fig. S3

Guo *et al.*

A

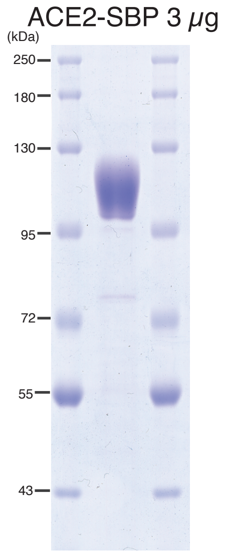

B

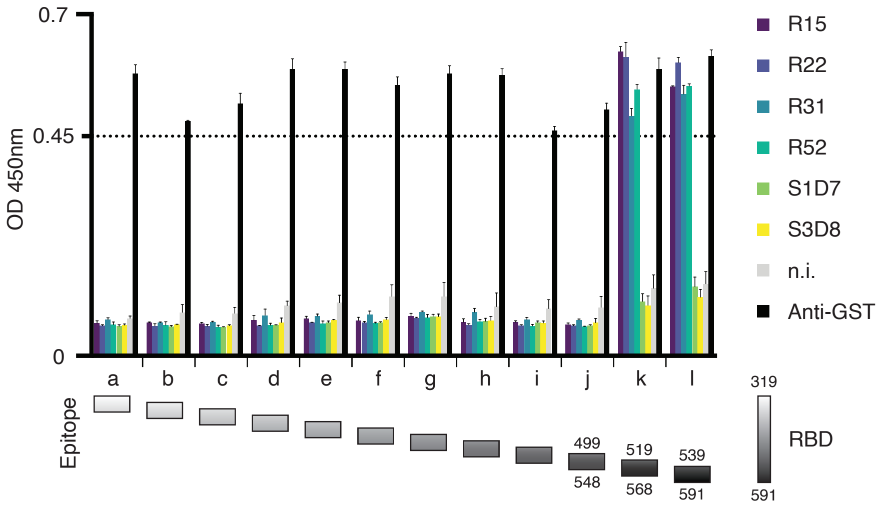

C

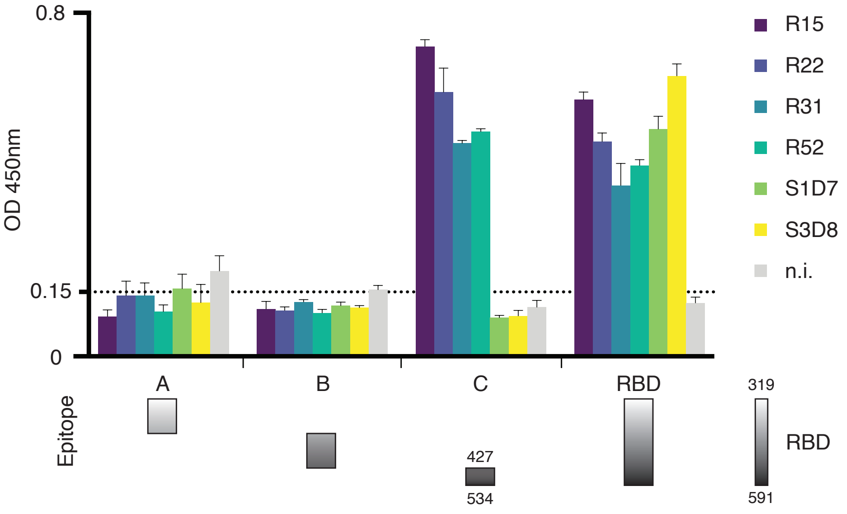

D

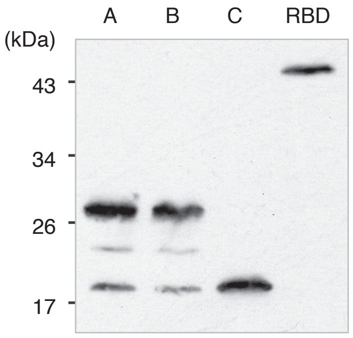

Fig. S4

Guo *et al.*

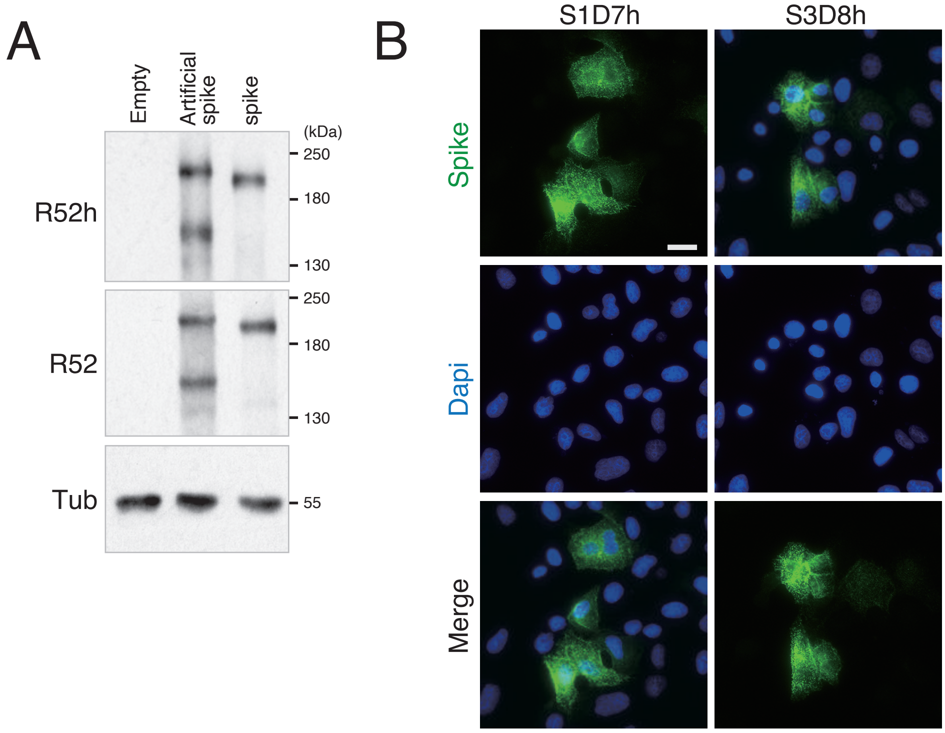

Table S1. Monoclonal antibodies neutralizing SARS-CoV-2 infection.

Clone	Host species	Cell line	Virus titer			IC_{50} (ng/ml)	Reference
			TCID50	PFU	FFU		
BD-368-2	Human	Vero	-	100	-	15	Cao *et al.*, Cell (17)
0304-3H3		VeroE6	100	-	-	110	Chi *et al.*, Science (19)
1B07*		VeroE6	-	-	100	37	Hassan *et al.*, Cell (28)
P2C-1F11		VeroE6	-	-	600	15	Ju *et al.*, Nature (22)
S309		VeroE6	-	-	100	79	Pinto *et al.*, Nature (26)
CC6.33		HeLa-ACE2	-	150	-	39	Roger *et al.*, Science (27)
CB6		VeroE6	100	-	-	36	Shi *et al.*, Nature (25)
47D11		VeroE6	500	-	-	570	Wang C *et al.*, Nat Commun (20)
B38		Vero	100 / 200	-	-	177 / 1,967	Wu *et al.*, Science (21)
COV2-2196		VeroE6	-	-	100	15	Zost *et al.*, Nature (29)
S1D7	Mouse	VeroE6/TM2	1500	-	-	405.2	This paper
S3D8		VeroE6/TM2				139	

*1B07 is a chimeric monoclonal antibody which combines mouse Fv and human Fc.

Table S2. Protein sequence of antibody variable regions.

Clone	Isotype	Chain	FR1	CDR1	FR2	CDR2	FR3	CDR3	FR4
R15	IgG1k	heavy	QIQLVQSGPELKKPGETVKISCKAS	GYTFTKYG	INWVKQAPGKDLKWMGW	INTYTGEP	AYGDDFKGRFAFSLETSTNTAYLQINNFKNEDTATYFC	ARNGAMDY	WGQGTSVTVSS
		kappa	DVVMTQNPLTLSVTIGQPASISCKSS	QSLLDSDGKTY	LIWLLQRPGQSPKRLIS	LVS	KLDSGVPDRFTGSGSGTDFTLKISRVEAEDLGVYYC	WQGTHFPGT	FGGGTKLEIK
R22	IgG2ak	heavy	EVQLQQSGPELVRPGASVKISCKTS	GYTFTEYI	IHWVKQSHGKSLEWIGG	IDPNNGDT	SYNQKFKGKASLTVDKSSSTAYMELRSLTSDDSAVYYC	ARLDFDY	WGQGTTLTVSS
		kappa	DIVMTQSHKFMSTSVGDRVSITCKAS	QDVRTA	VAWYQQKPGQSPKLLIY	WAS	TRYTGVPDRFTGSGSGTDYTLTISSVQAEDLALYYC	QQHDSTPLT	FGAGTKLELK
R31	IgG2bk	heavy	QVQLQQSGAELVRPGTSVKISCKAS	YTFTNYW	LAWVKQRPGHGLEWIGD	IYPGGAYT	NFNEKFKGKATLTADTSSSTAYMQLSSLTSEDSAVYFC	ARDGHYGYAMDY	WGQGTSVTVSS
		kappa	DIVMTQAAFSNPVTLGTSASISCRSS	KSLLHSNGITY	LYWYLQKPGQSPQLLIY	QMS	DLASGVPDRFSSSGSGTDFTLRISRVEAEDVGVYYC	AQNLELPRT	FGGGIKLEIK
R52	IgG1k	heavy	QIQLVQSGPELKKPGETVKISCKAS	GYTFTNYG	MNWVKQAPGNGLRWMGW	INTYTREP	SYAADFKGRFAFSLETSARAAYLQINNLKNEDTATYFC	ARNGAMDY	WGQGTSVTVSS
		kappa	DVVMTQTPLTLSVTFGQPASISCKSS	QSLLDSDGKTY	LIWLLQRPGQSPKRLIS	LVS	KLDSGVPDRFTGSGSGTDFTLKISRVEAEDLGVYYC	WQGTHFPGT	FGGGTKLEIK
S1D7	IgG2ak	heavy	QVQLQQSGAELVKPGASVKLSCKAS	GYTFTSYY	IYWMKQRPGQGLEWIGE	INPNNGGT	NFNEKFKSKATLTVDKSSSTAYMQLSSLTSEDSAVYYC	TRGHSDY	WGQGTTLTVSS
		kappa	DIVLTQSPASLAVSLGQRATISCRAS	ESVEYYGTGL	MQWYQQKPGQPPKLLIY	AAS	NVESGVPARFSGSGSGTDFSLNIHPVEEDDIAMYFC	QQTRKVPYT	FGGGTKLEIK
S3D8	IgG2ak	heavy	QVQLQQSGAELVKPGASVKLSCKAS	GYTFTSYY	MYWMKQRPGQGLEWIGE	INPSNGGT	NFNEKFKSKATLTVDKSSSTAYMQLSSLTSEDSAVYYC	TIFITRDAMDY	WGQGTSVTVSS
		kappa	QIVLTQSPAIMSASPGEKVTLTCSAS	SSVSSSY	LFWYQQKPGSSPKLWIY	TTS	NLASGVPARFSGSGSGTSYSLTISSMEAEDAASYFC	HQWSSYPPT	FGAGTKLELK

FR: frame region, CDR: complementarity determining region

Supplemental Figure Legends

Figure S1

A. Recombinant spike glycoproteins were treated with HRV3C protease to remove SBP-tag before immunizing mice.

B. Clone R52 showed the highest performance on western blotting among our antibodies and detected even 0.08 ng SΔTM glycoprotein.

Figure S2

A. Quantification of signal intensity of spike glycoprotein immunoprecipitated by monoclonal antibodies. S1, S1D7; S3, S3D8; ni, non-immune IgG. Error bars indicate standard deviation (n=3).

B. Monoclonal antibody clones S1D7 and S3D8 maintain high efficiency even in the presence of 0.1% SDS. S1, S1D7; S3, S3D8; ni, non-immune IgG; In, input.

C. Immunofluorescence (IF) staining of spike glycoprotein expressed in HeLa cells with all six monoclonal antibodies. S1D7 and S3D8 showed higher performance in IF. Images were captured using a Keyence BZ-X810 fluorescence microscope. Scale bar, 200 μm.

D. Expression of SARS-CoV-2 M protein in 293T cells. 293T cells were transfected with plasmid encoding SARS-CoV-2 M protein, followed by WB using anti-flag antibody.

E. Hela cells were transfected with plasmids encoding SARS-CoV-2 spike and M proteins, followed by IF using anti-spike (S1D7) and anti-flag antibodies. Scale bar, 20 μm.

Figure S3

A. ACE2-SBP protein was purified from the culture supernatant of Expi293F cells transfected with a plasmid encoding ACE2-SBP.

B. ELISA using monoclonal antibodies against small overlapping RBD segments. Clone R15, R22, R31, and R52 recognized spike protein RBD; a.a. 519-568 and a.a. 539-591, but not a.a. 499-548, indicating a continuous epitope within overlapping a.a. 549-568. Clone S1D7 and S3D8 failed to recognize all small overlapping RBD segments. Error bars indicate standard deviation (n=3).

C. ELISA using monoclonal antibodies against 6×His-tagged RBD segments secreted in the culture medium of 293T cells. Clone R15, R22, R31, and R52 recognized spike protein RBD segment C, which contains continuous epitope a.a. 549-568. Clone S1D7 and S3D8 failed to recognize all RBD segments, suggesting that they require an intact RBD tertiary structure to bind spike protein. Error bars indicate standard deviation (n=3).

D. 6×His-tagged RBD segments were secreted in the culture medium of 293T cells and detected by western blotting.

Figure S4

A. R52h is applicable for WB. Lysates of 293T cells expressing artificial spikes carrying T4 foldon or wild-type spike glycoproteins were separated by SDS-PAGE, followed by WB using human/mouse chimeric antibody R52h which was secreted by 293T cells.

B. S1D7h and S3D8h are applicable for IF. Spike glycoprotein expressed in HeLa cells was stained with human/mouse chimeric antibody S1D7h or S3D8h which were secreted by 293T cells. Scale bar, 20 μm.

Detailed Experimental Procedures

Expression and purification of proteins in human cells

Synthetic DNA sequences encoding SARS-CoV-2 spike protein ectodomain (SΔTM, residue 1-1208; strain Wuhan-hu-1; GenBank: QHD43416.1) and RBD (residue 319-591; strain Wuhan-hu-1; GenBank: QHD43416.1) fused with an N-terminal signal peptide, a C-terminal trimerization motif, an HRV3C cleavage site, an SBP purification tag, and an 8xHis-tag were inserted into pEFx mammalian expression vector. S1/S2 (682-RRAR-685) and S2' (986-KV-987) cleavage sites of spike protein were mutated (682-GSAS-685, 986-PP-987, respectively) to prevent protease cleavage. The codon composition of DNA fragments was optimized and synthesized for protein expression in human cells (FASMAC). Full-length spike, human ACE2-SBP (residue 1-708; NP_001358344), and ACE2-FLAG were synthesized and cloned into pcDNA3.4 vector (Thermo Fisher). Recombinant proteins were prepared by Expi293 Expression System (Thermo Fisher) according to the manufacturer's instruction. They were secreted into culture medium supernatant of the Expi293F cells, and then affinity purified by Streptavidin Sepharose High Performance (Cytiva). Purification tags were removed by treating recombinant proteins with HRV3C protease (TaKaRa) and cOmplete™ His-tag Purification Resin (Roche). Purity and glycosylation of recombinant proteins were examined by PNGase F (N-Zyme Scientifics) treatment followed by SDS-PAGE and Coomassie staining. The containment measures for the living modified organisms in all experiments were confirmed by the Ministry of Education, Culture, Sports, Science and Technology of Japan on April 27, 2020.

Expression and purification of proteins in *E. coli*

The DNA sequence encoding SARS-CoV-2 spike protein RBD (residue 410-580; strain Wuhan-hu-1; GenBank: QHD43416.1) was amplified from a nasopharyngeal swab of a patient treated in the Keio University Hospital, and in-framed inserted into pGEX-5X-1 and pMAL-c2G *E. coli* expression plasmid, downstream of GST-tag and MBP-tag encoding sequence respectively. Sample collection from a patient is approved by Keio University Bioethics Committee with the number 20200063 and abide by the Declaration of Helsinki Principles. Recombinant proteins were expressed in overnight 16ºC cultured BL21(DE3)pLysS competent cells transformed by corresponding vector under induction of 1 mM Isopropyl β-D-1-thiogalactopyranoside (IPTG). MBP-tagged RBD was affinity purified by Amylose Resin (NEB) according to manufacturer's instructions; GST-tagged RBD was affinity purified by Glutatione Sepharose 4B (Cytiva) according to manufacturer's instructions. Purity of purified recombinant proteins were examined by SDS-PAGE followed by Coomassie staining.

Cell cultures

The mouse myeloma cell line SP2/0-Ag14 (RCB0209) was provided by the Riken Bioresouces Center (Tsukuba, Japan). The cells were cultured in RPMI 1640 (Nissui) supplemented with 10% heat-inactivated calf serum (Biowest) and 1 ng/mL recombinant human interleukin 6 (IL-6, PeproTech). HeLa and 293T cells were cultured in DMEM (Nacalai tesque) with 10% fetal bovine serum (Biowest). We maintained hybridoma clones against spike glycoproteins in Hybridoma Serum-free Medium (FUJIFILM Wako) supplemented with 1 ng/mL IL-6.

Production of monoclonal antibodies

BALB/c mice were immunized twice in 3-week intervals, with the second immunization serving as a booster. Mice were injected intraperitoneally with 100 μL chyle containing 10-50 μg antigen prepared with TiterMax Gold adjuvant (Sigma-Aldrich) according to the manufacturer's instructions. Four days after boosting, splenocytes of immunized mice were collected by grinding the spleens in RPMI 1640 medium. Splenocytes (1×10^8) were immediately mixed with 5×10^7 SP2/0 myeloma cells and fused using an electro cell fusion generator ECFG21 (NepaGene) according to the manufacturer's instructions. After fusion, cells were cultured in HAT medium (RPMI 1640 supplemented with 10% calf serum containing HT supplement (Gibco) and 0.4 μM aminopterin (Sigma-Aldrich)) for 10 days to select hybridomas. Hybridomas were subsequently screened by ELISA, in which RBD glycoproteins were generated from the Expi293F expression system. We performed western blotting and immunoprecipitation for further screening and subjected to monoclonization by serial dilution. For antibody production, monoclonal hybridomas were cultured in Hybridoma Serum-Free Medium (FUJIFILM Wako) supplemented with IL-6. Monoclonal antibodies were purified from hybridoma culture supernatants using Thiophilic-Superflow Resin (Clontech) or Ab-Capcher MAG2 (ProteNova) according to the manufacturer's instructions. The isotype of antibodies was determined using the IsoStrip Mouse Monoclonal Antibody Isotyping Kit (Roche).

Western blotting and immunoprecipitation

SΔTM and RBD glycoproteins were resolved on SDS-PAGE and transferred onto a nitrocellulose membrane (Amersham Protran, GE Healthcare). Lysates of 293T

cells transfected with plasmids encoding full-length spike glycoproteins was also separated by SDS-PAGE for WB. The membrane was blocked in 1% nonfat skim milk and then incubated in 1 μg/mL anti-spike antibodies for 1h at room temperature. After three times washing in PBS-T (0. 1% Tween 20), the membrane was incubated in 1:5000 dilution of the peroxidase-conjugated sheep anti-mouse IgG secondary antibody (MP Biomedicals) for 30 min at room temperature. Signals were detected using ECL Western Blotting Detection Reagents (GE Healthcare).

For immunoprecipitation assay, 1 μg of purified antibodies was conjugated to 10 μl Dynabeads Protein G (Thermo Fisher) for 30 min at room temperature, followed by washing twice in IP buffer (20 mM Tris-HCl(pH 7.4), 150 mM NaCl, 0.1% NP-40). Antibody conjugated beads were incubated with 100 ng SΔTM in 50 μl IP buffer for 2 hours at room temperature. Beads were washed three times in IP buffer and eluted with SDS-PAGE loading dye at 95ºC for 5 min. Immunoprecipitation of SΔTM was examined by SDS-PAGE followed by western blotting using antibody R52.

Immunofluorescence

Before performing immunofluorescence, HeLa cells seeded on cover glasses were transfected with plasmids encoding full length SARS-CoV-2 spike protein for 2 days using Lipofectamine 2000 (Thermo Fisher). Cells were fixed with 2% formaldehyde in PBS for 10 min at room temperature, washed in PBS-T once, and permeabilized with 0.1% Triton X-100 in PBS for 10 min at room temperature. Cells were blocked by 1% non-fat skim milk in PBS-T for 10 min, then incubated with 0.5 μg/mL antibody for 1 h at room temperature. After three times wash in PBS-T, cells were incubated in 1:500 diluted Alexa Fluor 488 conjugated goat anti-mouse IgG secondary antibody (Thermo

Fisher) and 1 μg/mL DAPI solution for 30 min at room temperature. The cover glasses were mounted with Prolong Glass Antifade Mountant (Thermo Fisher) overnight at room temperature before observing. The fluorescence images were taken with Keyence BZ-X810 fluorescence microscope and Olympus FV3000 confocal laser scanning microscope.

ELISA of antibody binding to SARS-CoV-2 spike protein

Nunc MaxiSorp™ flat-bottom 96-well plates (Thermo Fisher) were coated with 170 ng SΔTM in 50 μl PBS overnight at 4ºC, then blocked at room temperature for 1 hour by applying 200 μl of 3.75% BSA in PBS-T. Monoclonal antibodies starting from 100 μg/mL were four-folds serial diluted with blocking buffer to 12 gradients and incubated with plates for 1 hour at room temperature, followed by incubation with horseradish peroxidase (HRP) conjugated sheep anti-mouse secondary antibody (MP Biomedicals) 1:5000 diluted in blocking buffer for 30 min at room temperature. Plates were incubated for 15 min at room temperature with 1-Step Turbo TMB-ELISA Substrate Solution (Thermo Fisher), then terminated with equal volume of 1M phosphoric acid. Signal was quantified by measuring absorbance at 450 nanometer using iMark Microplate Absorbance Reader (Bio-Rad Laboratories). Half-maximum effective concentration (EC_{50}) was calculated by non-linear regression analysis of absorbance curve.

Epitope mapping of monoclonal antibody

For small overlapping RBD segments, the respective DNA sequences encoding spike protein residues 319-368, 339-388, 359-408, 379-428, 399-448, 419-468, 439-488, 459-508, 479-528, 499-548, 519-568, and 539-591 were subcloned from full-length spike

protein coding sequence, and in-framed inserted into pGEX-5X-1 *E. coli* expression plasmid, downstream of GST-tag encoding sequence. Recombinant proteins were expressed in 3 mL of 37 ºC overnight cultured BL21(DE3)pLysS cells transformed by the corresponding plasmid under induction of MagicMedia *E. coli* Expression Medium (Thermo Fisher). Cells were washed once and lysed in 250 μL PBS by sonication, 5 times diluted with PBS, then applied 50 μL per well in a 96 well plate as antigen in ELISA for epitope mapping.

For RBD segments secreted by 293T cells, the respective DNA sequences encoding spike protein residue 319-426, 427-534, and 535-591 were subcloned from full-length spike protein coding sequence, and in-framed inserted into pEFx mammalian expression plasmid, downstream of sequence encoding an N-terminal signal peptide followed by a 6×His-tag, an SBP-tag, and an HRV3C cleavage site. Recombinant proteins were expressed by transfecting 293T cells at a starting number of 1×10^6 cells in a 6 cm culture dish with the corresponding plasmid using Lipofectamine 2000 (Thermo Fisher). Three days after transfection, culture supernatants were collected and filtered by 0.45 μm filter, and then 50 μL of each antigen was applied for epitope mapping per well in ELISA.

ACE2-binding inhibition assay

For spike pull-down assay, SΔTM glycoprotein was incubated with 1 μg anti-spike antibody in 50 μl binding buffer (PBS supplemented with 0.1% NP-40) at room temperature for 1 hour, then 3 μg of ACE2-SBP recombinant protein was applied the reaction for 1 hour. The ACE2-SBP was pull-down by 10 μl Dynabeads M-270 Streptavidin (Thermo Fisher) for 30 min at room temperature, followed by washing twice with binding buffer and elution with SDS-PAGE loading dye at 95ºC for 5 min. ACE2-

Spike binding inhibition was examined by SDS-PAGE, followed by WB using antibody R52.

For bead-based neutralization assay, 20 μl of Streptavidin beads were incubated with 4 μg of RBD-SBP in 100 μl of TBSTx (TBS supplemented with 1% TritonX-100) overnight at 4°C with shaking. After washing, beads were incubated with diluted antibodies for 20 min at 4°C, washed, incubated with 4 μg/mL of ACE2-FLAG for 20 min at 4°C, washed, and incubated with an anti-DYKDDDDK antibody conjugated with APC fluorophore (MBL) for 20 min at 4°C. After the final wash, the mean fluorescence intensity (MFI) of beads was analyzed by a FACS Verse (BD). The relative MFI of beads was calculated by normalization using the MFI of beads incubated with non-immune mouse IgG.

Virus neutralization assay

SARS-CoV-2 virus (obtained from the National Institute of Infectious Diseases) was prepared from culture fluids harvested from infected VeroE6/TMPRSS2 cells (JCRB Cell Bank, JCRB1819) (41). The virus titer was 3×10^7 $TCID_{50}$/mL. The virus solution containing 1500 $TCID_{50}$ was incubated with each antibody at concentrations of serial threefold dilutions starting from 5 μg/mL. After incubating at room temperature for 1 h, the antibody-treated virus solution was mixed with VeroE6/TMPRSS2 cells in glass-bottom 96-well plates. At 7 h post-infection, cells were fixed in 4% PFA and subjected to indirect immunofluorescence assays using S1D7 antibody as described above. The number of infected cells were imaged and analyzed using ArrayScan (Thermo Fisher). Mouse anti-FLAG M2 antibody (Sigma) was also used as a control. Experiments with SARS-CoV-2 were performed in a biosafety level 3 (BSL3) containment laboratory at

University of Tsukuba.

Sequencing of antibody variable regions

Sequencing of antibody variable regions was carried out as described previously (56). Total RNA was extracted from the hybridoma cells using ISOGEN (NIPPON GENE) according to the manufacturer's instructions. 5' RACE (rapid amplification of 5' cDNA ends) was performed by the SMARTscribe Reverse Transcriptase kit (Clontech) in the presence of a Template-switch oligonucleotide (5'-AAGCAGTGGTATCAACGCAGAGTACATrGrGrG-3') containing three riboguanines (rGrGrG) at its 3' end. First-strand cDNA for heavy chain and kappa chain were synthesized using reverse RT primers, mIGHG_RT (5'-AGCTGGGAAGGTGTGCACAC-3') and mIGK_RT (5'-TTGTCGTTCACTGCCATCAATC-3'), respectively. cDNA for the heavy chain was amplified by PCR using universal forward primer, ISPCR (5'-AAGCAGTGGTATCAACGCAGAG-3'), and mIGHG_PCR primer (5'-GGGATCCAGAGTTCCAGGTC-3'), followed by Sanger sequencing. For the kappa chain, cDNA was amplified by PCR using ISPCR and mIGK_PCR primers (5'-ACATTGATGTCTTTGGGGTAGAAG-3'), and cloned into an *Eco*R V site of the pCAGGS-3Flag vector, followed by Sanger sequencing. The cloning step is required to sequencing the kappa light chain since a hybridoma cell line derived from myeloma SP2/0-Ag14 expresses an endogenous and aberrant kappa mRNA (GenBank Accession No. M35669) (57).

Expression of chimeric human/mouse anti-spike antibodies

The kappa and heavy chain variable regions were amplified and individually cloned by NEBuilder (NEB) into pcDNA3.4 carrying the constant regions of human IgG1. Recombinant antibodies were prepared by Expi293 Expression System (Thermo Fisher) according to the manufacturer's instruction. They were secreted into culture medium supernatant of the Expi293F cells, and then affinity purified by Ab-Capcher MAG2 (ProteNova). Recombinant antibodies were also secreted from commonly used 293T cells, into which the plasmids for heavy and light chains were co-transfected at a ratio of 1:2 by polyethylenimine. The culture supernatants containing antibodies were available for WB (R52h) and IF (S1D7h), detecting spike glycoprotein expressed in 293T or HeLa cells. The peroxidase-conjugated goat anti-human IgG (H+L) (Jackson ImmunoResearch) and Alexa Fluor 488 conjugated goat anti-human IgG (H+L) (Thermo Fisher) were used as secondary antibodies for WB and IF, respectively.
